# *Staphylococcus aureu*s secreted lipases do not inhibit innate immune killing mechanisms

**DOI:** 10.12688/wellcomeopenres.16194.2

**Published:** 2021-06-25

**Authors:** Fiona Sargison, Mariya I Goncheva, Joana Alves, Amy Pickering, J Ross Fitzgerald

**Affiliations:** 1 The Roslin Institute, Edinburgh, UK

**Keywords:** Staphylococcus, lipase, neutrophils, macrophages, innate immune cells

## Abstract

**Background:** *Staphylococcus aureus* causes an array of diseases in both humans and livestock. Pathogenesis is mediated by a plethora of proteins secreted by *S. aureus*, many of which remain incompletely characterised. For example, *S. aureus* abundantly secretes two isoforms of the enzyme lipase into the extracellular milieu, where they scavenge upon polymeric triglycerides. It has previously been suggested that lipases may interfere with the function of innate immune cells, such as macrophages and neutrophils, but the impact of lipases on phagocytic killing mechanisms remains unknown.

**Methods:** We employed the epidemic *S. aureus* clone USA300 strain LAC and its lipase deficient isogenic mutant, along with recombinant lipase proteins, in *in vitro* experimental infection assays. To determine if lipases can inhibit innate immune killing mechanisms, the bactericidal activity of whole blood, human neutrophils, and macrophages was analysed. In addition, gentamycin protection assays were carried out to examine the influence of lipases on *S. aureus* innate immune cell escape.

**Results:** There were no differences in the survival of *S. aureus* USA300 LAC wild type and its lipase-deficient isogenic mutant after incubation with human whole blood or neutrophils. Furthermore, there was no detectable lipase-dependent effect on phagocytosis, intracellular survival, or escape from both human primary and immortalised cell line macrophages, even upon supplementation with exogenous recombinant lipases.

**Conclusions:** *S. aureus* lipases do not inhibit bacterial killing mechanisms of human macrophages, neutrophils, or whole blood. These findings broaden our understanding of the interaction of *S. aureus* with the innate immune system.

## Introduction

The Gram-positive bacterium *Staphylococcus aureus* is the cause of an array of nosocomial and community-acquired infections. To be a successful pathogen, *S. aureus* must evade killing by the innate immune system which it does via a large number of secreted factors. Within the *Staphylococcus* genus, a lipase-encoding gene ( *lip1*) is present in at least 12 species, and a second lipase gene is present in *S. aureus* ( *lip2)* and *S. epidermidis (gehD)* ^1, 2^. *S. aureus* lipases are glycerol-ester hydrolases that cleave triglyceride lipids, resulting in the release of glycerol derivatives and free fatty acids ^1^. Lipase 1 has an affinity for short-chain fatty acids, whereas lipase 2 has no bias towards chain length ^1^. Transcription of lipase genes is regulated by the accessory gene regulator ( *agr)* two component system, leading to the expression of a pre-pro-lipase precursor that is secreted into the extracellular milieu ^1, 3^. The catalytic activity of lipases is regulated through downstream processing by the secreted zinc metalloprotease, Aur, which proteolytically cleaves the pre-pro precursor enzyme resulting in the mature, active form of the enzyme ^4^. The activity of the mature lipase is governed by a catalytic triad, which cleaves glycerol-ester bonds through a serine hydrolase mechanism ^1, 2^. Lipases have been reported to account for approximately 20% of the total *S. aureus* secretome, but our understanding of the role of lipases in host-pathogen interactions is limited ^5^. It has been shown that 80% of clinical isolates from both systemic and localised *S. aureus* infections exhibit lipolytic activity, and patients typically test positive for anti-lipase IgG in serum ^6, 7^. Lipases have further been attributed to the formation of biofilm, which subsequently confers resistance to toxic polyamines thus promoting bacterial persistence ^3, 8, 9^. Previous studies have further demonstrated that lipases can produce free-fatty acids from host lipid metabolites, such as low-density lipoproteins, which subsequently are incorporated into the lipid moieties of *S. aureus* ^10^. The incorporation of lipoprotein particles has been shown to render the bacterium resistant to the antimicrobial drug triclosan, which is commonly used in the treatment of *S. aureus* infection ^10^.

In a previous study, human granulocytes were treated with *S. aureus* lipases resulting in the loss of microvilli, projections, and pseudopodia on their surface suggesting a potential impact on phagocytosis or neutrophil extracellular trap (NET) formation ^11, 12^. More recently, it was demonstrated that lipase 2 interferes with macrophage signalling, which subsequently diminishes the downstream pro-inflammatory response ^13^. Specifically, lipase 2 inactivates *S. aureus* secreted lipoproteins, which are a major pattern-associated molecular pattern recognised by Toll-like receptor 2 (TLR2) in response to *S. aureus* infection ^13^.

Macrophages are equipped with an array of pathogen recognition receptors and, alongside a role in modulation of cellular signalling, are professional phagocytes that aid in the clearance *S. aureus* ^14, 15^. However, studies have shown that once entrapped within the macrophage phagolysosome, *S. aureus* can subvert killing mechanisms and persist for several days ^16, 17^. The subsequent death of the macrophage through membrane blebbing and caspase-3 activation results in the release of viable bacteria, promoting intra-host dissemination in a Trojan horse-like system ^14, 17, 18^.

Here, we tested the hypothesis that lipases can interfere with the antibacterial activity of whole blood, neutrophils and macrophages. We report that, despite their abundant secretion, lipases have no effect on killing, phagocytosis, intracellular survival or escape of *S. aureus* USA300 LAC.

## Methods

### Bacterial growth conditions

40% (v/v) glycerol stocks of both a wild type ( *S. aureus* USA300 WT) and an isogenic mutant ( *S. aureus* USA300 *Δlip1/Δlip2)* of the CC8 epidemic clone *S. aureus* USA300 LAC generated in a previous study were stored at -80°C. When required, stocks were sub-cultured onto tryptone soy agar (TSA, Oxoid CM131B) or cultured into tryptone soy broth (TSB, Oxoid CM129B) overnight at 37°C with agitation (200 rpm). The culture was diluted 1 in 100 in TSB and incubated, until exponential phase (OD _600_=0.6–0.8), as measured using an Amersham Biosciences Ultrospec 2100 pro spectrophotometer. For infection protocols, bacteria were washed in cell culture media and suspended at the required OD _600_.

### Lipase Mutant Strain Construction

Gene deletions of S. aureus were produced by allelic replacement, as described by Monk *et al.* ^19^. Briefly, 500-1000 base pair regions flanking the lipase 1 gene were amplified using primers (Lip1_A: GCGCGTCGACGCTGTGATGGTACTAAAGTTGC, Lip1_B; TAGAGGTGCTGACAATG, Lip1_C: TAGAGGTGCTGACAATGGGCATTTGGCAAGTGACGCCTAC, Lip1_D: GCGCGGTACCTGCTGTACCAAATGGAGACT) and spliced into pIMAY. The plasmid was transduced into recipient USA300 LAC Δlipase 2 (kindly provided by Dr David Heinrichs, University of Western Ontario, Canada). Deletion of the lipase 1 and lipase 2 genes were confirmed by both Sanger sequencing (Edinburgh Genomics, University of Edinburgh UK) and whole genome sequencing (MicrobesNG, Birmingham UK).

### Purification of recombinant lipase1 (rLip1) and 2 (rLip2)

Expression plasmid constructs pET156:: *lip1* or pET156:: *lip2* ^20^ were transformed into ClearColi ^®^ BL21 (DE3) electrocompetent cells (Lucigen, 60810-1) by electroporation, according to the manufacturer’s instructions. Cells were grown in LB Miller broth (Sigma, L3522-250G) to an OD _600_ of 0.6 and protein expression was induced with 1 mM isopropyl β-D-1-thiogalactopyranoside (IPTG, Formedium Ltd, IPTG025) for 4 h at 37°C, with agitation (200 rpm), before centrifugation and storage at -20°C.

Hexa-histidine tagged proteins were purified by immobilised metal affinity chromatography as described previously ^20^. Western blot analysis confirmed the presence of hexa-histidine tagged proteins at 76 kDa (1 in 10,000 monoclonal anti-poly His, α-diagnostics HISP12-HRP, in 8% (w/v) skimmed milk (Sigma, 70166-500G) in sterile phosphate buffered saline (PBS)). Primary antibody binding was detected using enhanced luminol-based chemiluminescent (ECL) western blotting substrate (GE Healthcare, RPN2232). For lipopolysaccharide (LPS) removal, 1 ml of Pierce high capacity endotoxin removal resin (Thermo Fisher Scientific, 88271) was used according to the manufacturer’s instructions and proteins were quantified using a bicinchoninic acid (BCA) assay (Merck millipore, 71285-3). To analyse recombinant protein lipolysis, a turbiometric assay was used following the methodology outlined previously ^21^. For each of the following experiments, 200 nM of recombinant lipase 1 (rLip1) or 2 (rLip2) was used, according to previous estimates of lipase secretion levels by *S. aureus* ^22^.

### Ethics statement

Human blood was obtained from healthy volunteers in syringes treated with anticoagulant citrate dextrose. Ethical approval for the collection of blood from anonymous donors was granted by the University of Edinburgh Research Ethics Committee. This study was reviewed by the University Of Edinburgh College Of Medicine Ethics Committee (2009/01) and subsequently renewed by the Lothian Research Ethics Committee (11/AL/0168). Written informed consent was received from all volunteers participating in the study.

### Bacterial killing by neutrophils

Neutrophils were purified from human blood using a ficoll gradient. Briefly, 10 ml of 1.077 g/mol ficoll paque plus (Fisher, 11778538) was gently layered onto 12 ml of 1.119 g/mol Histopaque plus (Sigma, 11191). Fresh human blood was diluted at a 1:1 ratio in Ca ^2+^ and Mg ^2+^ free PBS (Lonza, BE17-515F), then slowly pipetted onto the ficoll gradient prior to centrifugation for 20 min at 400 × *g* (without a brake). The neutrophil layer was collected, cells were centrifuged and erythrocytes lysed by osmotic shock. Cells were suspended in RPMI-1640 (Sigma, R5886), 0.05% (v/v) human serum albumin (Sigma, A9080-10ML) and 1% (v/v) GlutaMAX (Gibco, 35050-061) prior to use. 50 µl of 1.5 × 10 ^5^ colony forming units (CFU) of *S. aureus* USA300 WT or *S. aureus* USA300 *Δlip1/ Δlip2* bacterial cells were opsonised in 50 µl of 10% autologous human plasma for 15 min in a 96 well Cellstar U bottomed plate (Greiner Bio-One Inc, 650101) (37°C). Subsequently, 1.5 × 10 ^4^neutrophils (MOI=10) were incubated with the bacteria in the presence or absence of 200 nM rLip1 or rLip2. The plate was shaken at 600 rpm for 30 min at 37°C followed by cell lysis in 0.1% Triton X-100 (Sigma, P6416-100ML) and plated onto TSA using a modified Miles-Misra technique ^23^, whereby 10 µl of each 10-fold bacterial dilution was plated, followed by incubation overnight at 37°C and counting of colonies.

### Bacterial killing by whole blood

75 µl of whole blood was infected with 25 µl of 1. 5 × 10 ^5^ CFU of *S. aureus* USA300 WT and *S. aureus* USA300 *Δlip1/Δlip2* in the presence or absence of 200 nM rLip1, rLip2 or both in a 96 well Cellstar U bottomed plate for 1, 2 and 4 h at 37°C, with shaking at 200 rpm. Blood was lysed in 0.1% (v/v) TritonX-100 (Sigma), viable bacteria counts were determined with 10 µl of ten-fold bacterial dilutions in PBS onto TSA using a modified Miles-Misra technique ^23^ and incubated overnight at 37°C.

### Isolation of CD14 ^+^ monocytes

Monocytes were isolated from human whole blood following centrifugation at 1200 *x g* (no break) for 20 min. Buffy coats were combined and diluted with PBS and subsequently slowly pipetted over 15 ml of 1.199 g/mol ficoll paque plus (Sigma). A gradient was generated by centrifugation for 45 min at 200 *x g* (no break), in which the mononuclear cell layer was subsequently removed. Ficoll was removed by centrifugation for 10 min with 300 *x g,* and resuspension in PBS *.* CD14 ^+^ monocytes were collected using a MAC-LS column as per the manufacturer’s instructions (Miltenyi Biotec, 130-042-401).

### Macrophage differentiation

For THP1 differentiation into macrophages, 5 × 10 ^5^ THP1 cells were seeded in a 96-well Nunc flat bottomed plate in RPMI-1640 (Sigma), 10% (v/v) heat-inactivated foetal bovine serum (Gibco, 10270-106) and 1% (v/v) GlutaMAX (Gibco) in the presence of 200 nM phorbol 12-myristate 13-acetate (PMA, VWR P1585-1MG) for 3 d, before being left to rest for 1 d in media without PMA. For blood monocyte-derived macrophages, 5 × 10 ^5^ of purified human blood CD14 ^+^ cells were incubated for 5 d in 1:100 dilution of 10 ^4^ U/ml recombinant human colony-stimulating factor-1 (hCSF-1, provided by Prof. D. Hume) in media. On the 5 ^th^ day, cells were topped up with 25% complete medium containing 3 × the target concentration of hCSF-1 and cells were used at day 7.

### Gentamycin-protection assay

THP1 macrophages and blood-monocyte derived macrophages were infected at an MOI of 1 with bacteria suspended in fresh media (RPMI-1640 (Sigma), 10% (v/v) heat-inactivated foetal bovine serum (Gibco) and 1% (v/v) GlutaMAX (Gibco). Cells were centrifuged at 400 *x g* for 5 min and incubated for 1 h at 37°C, 5% CO _2_. For analysing internalised bacteria (phagocytosis), cells were subsequently incubated with 100 µg/ml gentamycin (Sigma, G1397-10ML) in cRPMI for 30 min. To analyse intracellular survival, cells were subsequently left in 20 µg/ml gentamycin in media and were incubated for a further 24 h at 37°C, 5% CO _2_. Finally, to analyse the escape of intracellular bacteria, cells were incubated for 24 h in antibiotic-free media at 37°C, 5% CO _2_. At each time point, corresponding to the degree of phagocytosis, bacterial intracellular survival, and bacterial escape from the macrophage, cells were lysed in 0.1% Triton X-100 in PBS for 5 min at room temperature, and viable cell counting by plating onto TSA as described above.

### Statistical methods

Statistical analysis was performed with GraphPad Prism 8 software (GraphPad, USA).

## Results

### Lipases do not inhibit *S. aureus* survival in human whole blood

**Figure 1.**
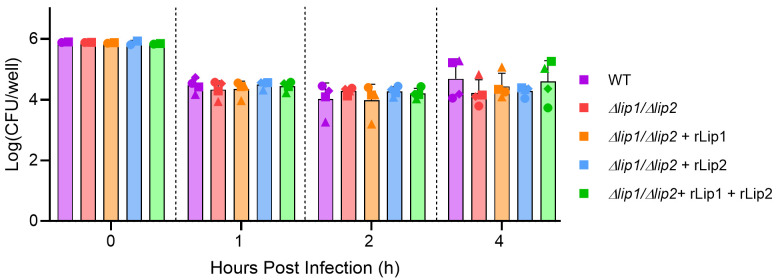
Lipases do not promote survival of *S. aureus* in human whole blood. Human whole blood was collected from healthy donors (each donor represented by a different symbol) and incubated with *S. aureus* USA300 WT or *S. aureus* USA300 *Δlip1/Δlip2,* alongside *S. aureus* USA300 *Δlip1/Δlip2* supplemented with 200 nM rLip1 and/or rLip2 for 0, 1, 2 and 4 h, at 37°C (with agitation). Each symbol represents the average of technical triplicates. Two-way ANOVA with Tukey's multiple comparisons. Bars show mean + SD, n=4.

Peripheral whole blood contains an array of innate immune components involved in the direct killing of *S. aureus* ^24– 28^. To evaluate if lipases can promote *S. aureus* survival in blood, human whole blood was incubated with *S. aureus* USA300 LAC ( *S. aureus* USA300 WT) or its isogenic mutant deficient in both lipase 1 and lipase 2 production ( *S. aureus* USA300 *Δlip1/Δlip2*) for 1, 2, and 4 h at 37°C. Concurrently, *S. aureus* USA300 *Δlip1/Δlip2* was also incubated with 200 nM of functionally active rLip1 and rLip2 (Extended Figure 1 ^29^). There was a 10-fold reduction in the number of recoverable bacteria in the first hour post-infection, followed by a stabilisation of the number of viable bacteria recovered up to 4 h, but there was no difference between the *S. aureus* USA300 WT and the lipase-deficient mutant or strains supplemented with recombinant lipase ( Figure 1 ^29^). Overall, these data indicate that lipases do not inhibit killing of *S. aureus* USA300 LAC in human whole blood.

### *S. aureus* lipases do not inhibit neutrophil bactericidal activity

**Figure 2.**
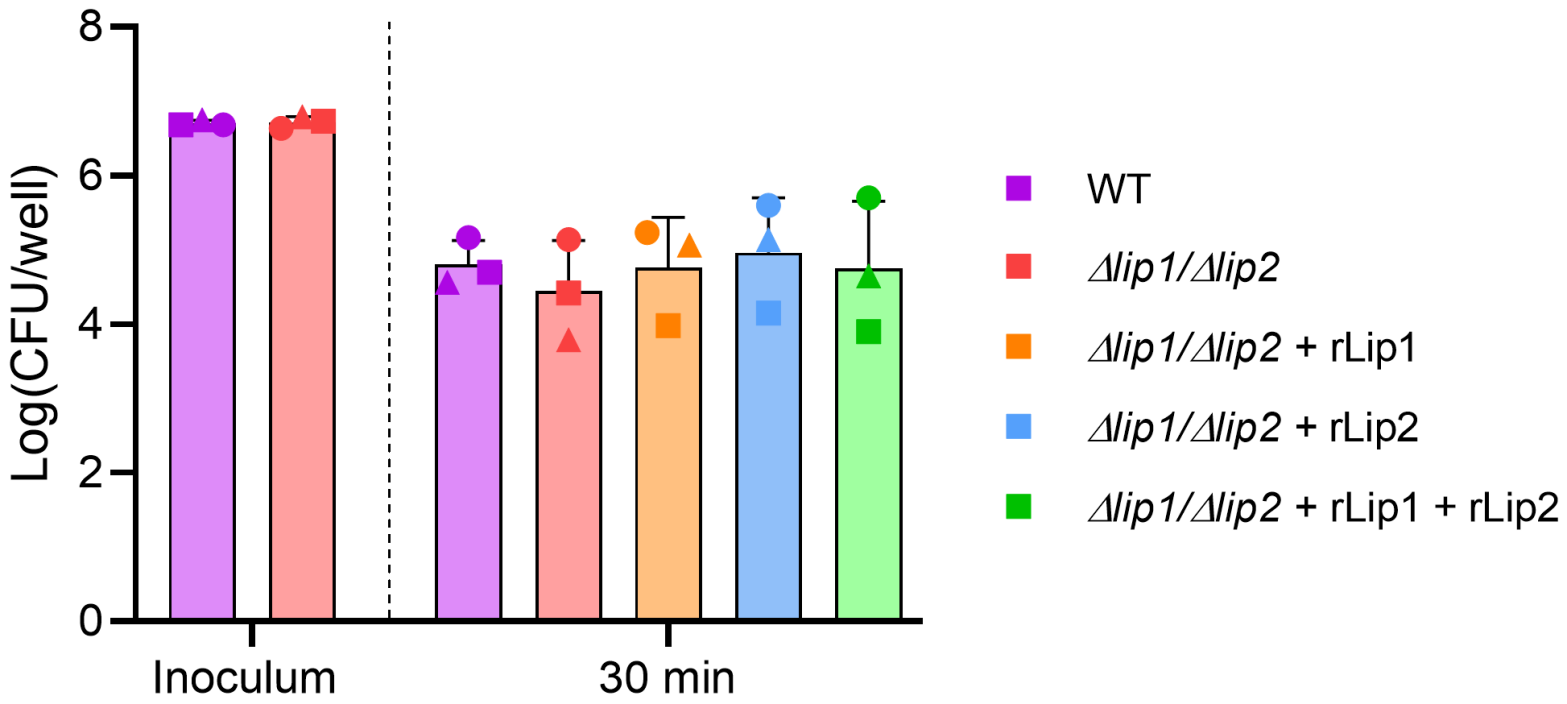
Lipases do not inhibit human neutrophil bactericidal activity. Human neutrophils were isolated from healthy donors (each donor represented by a different symbol) and incubated with plasma opsonised *S. aureus* USA300 WT or *S. aureus* USA300 *Δlip1/Δlip2* at an MOI of 10, in the presence or absence of 200 nM rLip1 and/or rLip2 for 30 min at 37°C (with vigorous agitation). Each symbol represents the mean of 5 technical replicates for an individual donor. One-way ANOVA with Tukey's multiple comparisons. Bars show mean + SD, n=3.

It was previously demonstrated that purified *S. aureus* lipases alter the phenotype of granulocytes, suggesting a possible impact on their function ^11, 12^. To establish if lipases can inhibit neutrophil killing of *S. aureus*, human neutrophils were isolated from fresh whole blood and incubated with opsonised *S. aureus* USA300 WT or *S. aureus* USA300 *Δlip1/Δlip2* for 30 min. As with whole blood, there was a 10-fold reduction in the number of viable bacteria after incubation with neutrophils, but viability between the *S. aureus* USA300 WT and the lipases-deficient strain did not differ ( Figure 2 ^29^). In addition, neutrophils were incubated with *S. aureus* USA300 *Δlip1/Δlip2* in the presence of exogenous recombinant lipases and there were no differences in the number of recovered viable bacteria between the tested conditions ( Figure 2 ^29^). Taken together, these data indicate that lipases do not inhibit neutrophil-mediated killing of *S. aureus* USA300 LAC.

### Lipases do not influence phagocytosis, intracellular survival or escape of *S. aureus* from macrophages

**Figure 3.**
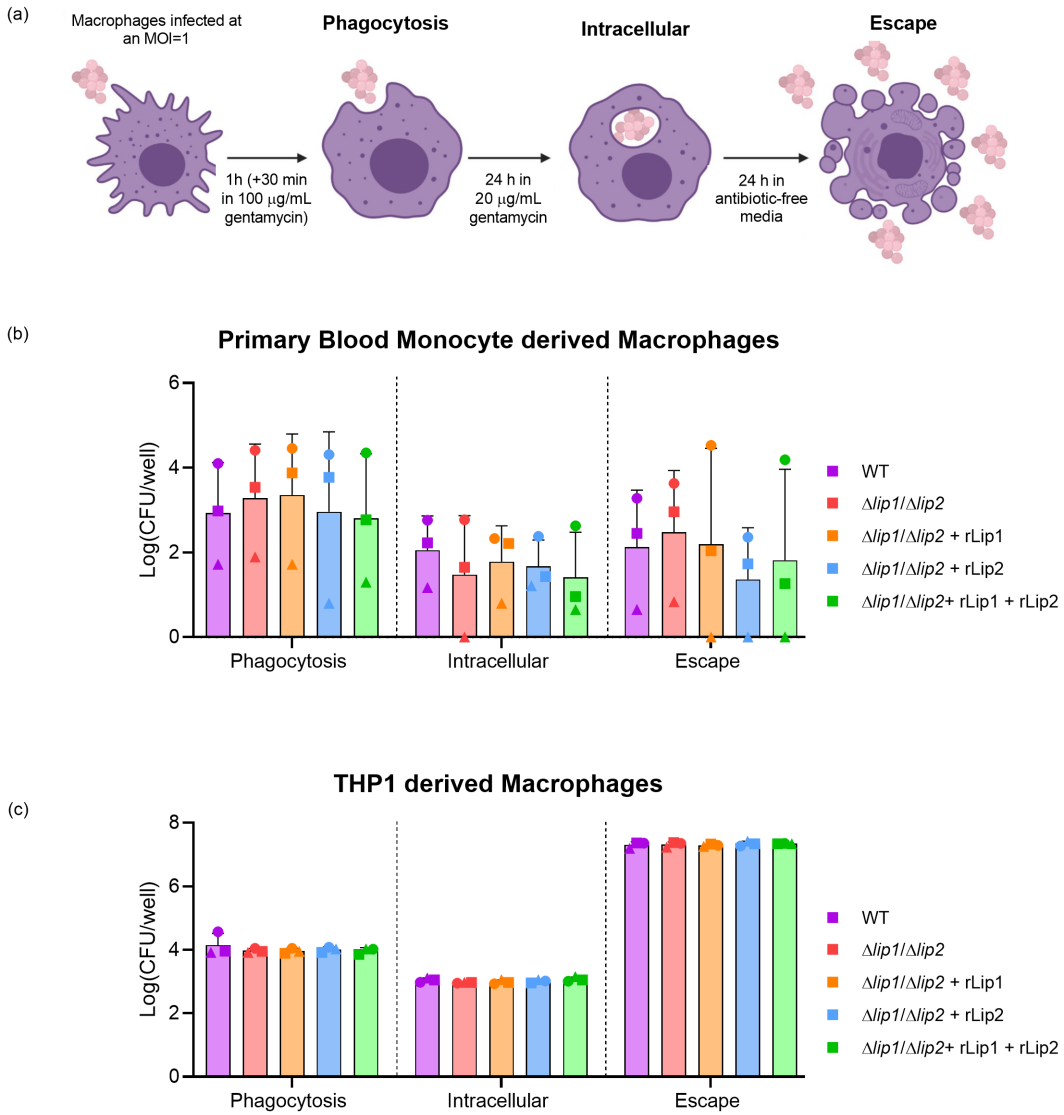
Lipases do not affect bacterial survival in human blood monocyte or THP1 derived macrophages. ( **a**) Schematic of the assay used to analyse the phagocytosis, intracellular proliferation and escape of *S. aureus* from within macrophages. ( **b**) Primary macrophages were differentiated from human blood monocytes isolated from 3 different healthy donors (represented by different symbols) and were incubated with *S. aureus* USA300 WT or *S. aureus* USA300 *Δlip1/Δlip2,* alongside *S. aureus* USA300 *Δlip1/Δlip2* supplemented with 200 nM rLip1 and/or rLip2 as per the schematic in Fig 4a, followed by plating and viable counting. ( **c**) THP1 macrophages were incubated with *S. aureus* USA300 WT or *S. aureus* USA300 *Δlip1/Δlip2,* alongside *S. aureus* USA300 *Δlip1/Δlip2* supplemented with 200 nM rLip1 and/or rLip2 following the schematic in Fig 4a. CFU analysis of phagocytosis, 24 h intracellular survival and 24 h escape was quantified for 3 technical replicates. Paired data was analysed using a two-way ANOVA, Tukey’s multiple comparisons. Bars show mean + SD, n=3.

Recently, it was demonstrated that lipolysis of *S. aureus* lipoproteins by lipase 2 facilitated the survival of *S. aureus* through the manipulation of macrophage cellular signalling ^13^. In addition, *S. aureus* can interfere with macrophage phagolysosomal killing, enabling intracellular persistence ^16^. To examine the capacity for *S. aureus* lipases to influence phagocytosis, intracellular survival, and escape from macrophages, primary human monocyte-derived macrophages were incubated with *S. aureus* USA300 WT or *S. aureus* USA300 *Δlip1/Δlip2* in the presence or absence of rLip1 or rLip2 ( Figure 3a ^29^). Considerable variation in the number of recovered bacteria was observed between technical replicates due to donor variability, but no significant lipase-dependent differences were observed ( Figure 3b ^29^). To further explore the effect of lipases on macrophage function, an immortalised cell line derived from human peripheral blood monocytes (THP1) cells was employed ^30^. PMA induces THP1 monocyte differentiation into adherent macrophages which represent a model of human monocyte-derived macrophages ^31^. *S. aureus* USA300 LAC infection of THP1 macrophages exhibited less variation between replicates when compared to primary cultures but no lipase-dependent differences in the number of bacteria recovered was observed ( Figure 3c ^29^). Together, these data indicate that lipases do not affect phagocytosis, survival or escape of *S. aureus* from human macrophages.

## Discussion

The importance of neutrophils in the initial response to *S. aureus* infection is well established ^25, 32^. Previously, Rollof *et al.,* demonstrated, using scanning electron microscopy, that supernatant-purified *S. aureus* lipases altered granulocyte morphology by denuding surface projections ^11^. As neutrophil phagocytosis is reliant on pseudopod extensions for ingesting bacteria, it was hypothesised that this phenotype could inhibit bactericidal activity ^25^. Furthermore, the release of extracellular DNA into the environment, through NETosis, could be impacted by lipase-mediated changes to the cellular membrane which could influence bacterial killing.

Here, we demonstrate that lipases do not inhibit direct killing of *S. aureus* mediated by human neutrophils, macrophages or whole blood *in vitro*. The findings are consistent with the findings of Nguyen *et al.,* who did not observe any differences in bacterial burden in the heart and liver in an *in vivo* murine sepsis model 24 h after infection with *S. aureus* USA300 WT LAC or an isogenic lipase-deficient mutant ^3^. These data suggest that lipases do not interfere with the initial clearance of *S. aureus* from the blood.

A recent study by Chen *et al.,* reported that lipases have no direct effect on initial bacterial clearance in the early stages of infection. However they demonstrated that after 48 h, there was an indirect effect of lipase 2 resulting in reduced pro-inflammatory cytokine release by macrophages ^13^. The authors found that *S. aureus* lipase 2 mediates cleavage of *S. aureus* lipoproteins, which are well characterised TLR2 ligands, resulting in increased bacterial burden by thwarting macrophage responses. 

Previously it has been shown that *S. aureus* virulence factors regulated by the *agr* quorum-sensing system are required for survival and escape of *S. aureus* from macrophages, including the zinc metalloprotease Aur which is responsible for the downstream activation of the catalytically active lipases ^16, 33, 34^. Here, we report that the *agr*-regulated lipases do not influence the survival of *S. aureus* in human monocyte-derived macrophages, although considerable donor specific variation was observed with primary cells. Data obtained using the THP1 macrophage cell line further support the finding that *S. aureus* lipases do not affect phagocytosis, intracellular survival or escape of *S. aureus* from human macrophages. The lack of an observable effect of lipases may reflect the fact that bacterial capture by macrophages is dependent on dynamic actin-rich protrusions, with negligible involvement of triglyceride lipids in the process ^35^.

## Conclusion

Overall, we report that *S. aureus* lipases do not directly impact on the killing mechanisms of neutrophils and macrophages. These data add to our understanding of *S. aureus* interactions with the innate immune system and the role of lipases in the pathogenesis of *S. aureus* disease.

## Data availability

### Underlying data

Edinburgh Datashare: *Staphylococcus aureus* secreted lipases do not inhibit innate immune killing mechanisms: Extended Figure 1. https://doi.org/10.7488/ds/2881 ^29^

This project contains the following underlying data:

- Validation of rLip1 and rLip2.xlsx (ClarioSTAR (BMG Labtech) readouts of both rLip1 and rLip2, alongside 400 nM BSA. Absorbance was measured at OD _495 _every 5 min for 20 h. Each experiment contained three technical repeats, n=3)
- Recombinant lipases Western blot, raw-unedited image.jpg (Raw gel image for Western presented in Extended Figure 1)

### Extended data

Edinburgh Datashare: Staphylococcus aureus secreted lipases do not inhibit innate immune killing mechanisms: Extended Figure 1. https://doi.org/10.7488/ds/2881 ^29^

- Extended Figure 1.docx

**Extended Figure 1: Functional characterisation of purified recombinant *S. aureus* lipase 1 and 2.** (a) Purification of recombinant lipase 1 and 2 was analysed using western blot, in which bands present at 76 kDa indicated the correct protein elution (detected by hexa-his tag, α-diagnostics HISP12-HRP). Page-Ruler ladder (furthest left well) shows the visible protein marker at 75 kDa. Measurement of lipolytic activity of recombinant protein 1 (rLip1) (b) and 2 (rLip2) (c). It was observed that both lipase 1 and 2 were functionally active enzymes which were able to cleave Tween-20 over a broad scope of concentrations. Indeed, it was also observed that lipase 2 was much more kinetically active in comparison to lipase 1, which could be attributed to its broader substrate range. Two-way ANOVA, Dunnets Multiple Comparisons against the BSA negative control, α=0.05, **** p<0.001. Each point shows mean + SD (Data represent a representative experiment, from three independent experiments).

Data are available under the terms of the Creative Commons Attribution 4.0 International license (CC-BY 4.0).
