# Supplementary material for: Staphylococcus aureus secreted lipases do not inhibit innate immune killing mechanisms
Source: Wellcome Open Res. 2021 Jun 25;5:286. Originally published 2020 Dec 9. [Version 2] doi: 10.12688/wellcomeopenres.16194.2 (PMC7871421; doi:10.12688/wellcomeopenres.16194.2)
Supplement: Peer review history

# Peer review: Reviewer response for version 2

**DOI:** 10.21956/wellcomeopenres.18284.r44668

**Published:** 2021-07-02

**Authors:** Meera Unnikrishnan

**Affiliations:** 1 Division of Biomedical Sciences, University of Warwick, Coventry, UK

Thank you for your responses.

Is the work clearly and accurately presented and does it cite the current literature?

Yes

If applicable, is the statistical analysis and its interpretation appropriate?

Yes

Are all the source data underlying the results available to ensure full reproducibility?

No source data required

Is the study design appropriate and is the work technically sound?

Partly

Are the conclusions drawn adequately supported by the results?

Yes

Are sufficient details of methods and analysis provided to allow replication by others?

Partly

Reviewer Expertise:

Host pathogen interactions: Intracellular S. aureus infections, Type VII secretion systems, C. difficile colonisation

I confirm that I have read this submission and believe that I have an appropriate level of expertise to confirm that it is of an acceptable scientific standard.

# Peer review: Reviewer response for version 1

**DOI:** 10.21956/wellcomeopenres.17782.r42337

**Published:** 2021-02-23

**Authors:** Meera Unnikrishnan

**Affiliations:** 1 Division of Biomedical Sciences, University of Warwick, Coventry, UK

This is a clearly written manuscript, investigating the role of the *S. aureus* lipase 1 and 2 in bacterial killing in whole blood, neutrophils and macrophages. This work is important as lipases are generally considered to impact innate cell functions, although effects on bacterial killing have not been directly assessed yet.

 In general experiments have been done well, and the main conclusions are valid. However a few technical clarifications are required.

 For the macrophage internalisation assays, when following bacterial replication by CFU, usually it is good to look at an earlier time point as by 24h there can be significant cell lysis induced by the WT. Have the authors checked the morphologies or the states of the macrophages during their experiments? 

 The bacterial 'escape' measurements from macrophages need some clarification. It is not clear if after the 1h infection the macrophages were treated with gentamicin to kill all the extracellular bacteria first prior to adding the antibiotic-free medium. This is essential to do in order to measure the escape of intracellular bacteria. It is also not clear if escape was quantified from the culture supernatants or from the the cell lysates. Quantifying bacterial escape is quite tricky, and should be preferably done at multiple time points after infection to get a clear picture. Counting from supernatants are not accurate as most bacteria settle down to the well by 24h forming microcolonies, and by 24h there is substantial cell lysis, so hard to deduce the 'escaped' population by measuring intracellular counts (by cell lysis). Finally, the authors may want to comment on why the 'escape' numbers are so high between the THP1 vs the primary cells.

Is the work clearly and accurately presented and does it cite the current literature?

Yes

If applicable, is the statistical analysis and its interpretation appropriate?

Yes

Are all the source data underlying the results available to ensure full reproducibility?

No source data required

Is the study design appropriate and is the work technically sound?

Partly

Are the conclusions drawn adequately supported by the results?

Yes

Are sufficient details of methods and analysis provided to allow replication by others?

Partly

Reviewer Expertise:

Host pathogen interactions: Intracellular S. aureus infections, Type VII secretion systems, C. difficile colonisation

I confirm that I have read this submission and believe that I have an appropriate level of expertise to confirm that it is of an acceptable scientific standard, however I have significant reservations, as outlined above.

# Response

**Published:** 2021-03-30

**Authors:** J Ross Fitzgerald

**Affiliations:** University of Edinburgh, UK

We would firstly like to thank you for your supportive comments regarding our manuscript and for your constructive review. Please find below the response to your individual questions below:

*For the macrophage internalisation assays, when following bacterial replication by CFU, usually it is good to look at an earlier time point as by 24h there can be significant cell lysis induced by the WT. Have the authors checked the morphologies or the states of the macrophages during their experiments?*

 At each time point, the macrophages were inspected under a light microscope. Indeed, after 24 hours of *S. aureus* intracellular survival, the macrophages appeared to be in an activated state based on their morphology but without significant cell lysis. Their phenotype was not dissimilar to observations of cell morphology at earlier time points in the experiment.

*The bacterial 'escape' measurements from macrophages need some clarification. It is not clear if after the 1h infection the macrophages were treated with gentamicin to kill all the extracellular bacteria first prior to adding the antibiotic-free medium. This is essential to do in order to measure the escape of intracellular bacteria. It is also not clear if escape was quantified from the culture supernatants or from the the cell lysates. Quantifying bacterial escape is quite tricky, and should be preferably done at multiple time points after infection to get a clear picture. Counting from supernatants are not accurate as most bacteria settle down to the well by 24h forming microcolonies, and by 24h there is substantial cell lysis, so hard to deduce the 'escaped' population by measuring intracellular counts (by cell lysis).*

 For measuring *S. aureus *escape we followed the procedure outlined in the schematic shown in Figure 3a. Cells were infected for 1 hour at an MOI of 1 followed by treatment with 100 µg/ml of gentamycin for 30 minutes to kill any extracellular bacteria. The cells were washed and then subsequently left for 24h in a low concentration of gentamycin (20 µg/ml) which does not penetrate the cells, however, was sufficient for killing any bacteria which had escaped due to cell lysis in this time. After 24 hours, the cells were washed again to remove any residual gentamycin and incubated in the antibiotic-free media for the 24 hour “escape” period. In preliminary data not shown, we analysed bacterial escape over a range of time points (ranging from 3-24 hours post removal of gentamycin media) and observed no differences in the recovered viable CFU in the presence and absence of lipase, and thus 24 hours was chosen as a representative time point. To quantify escape, 0.1% (v/v) TritonX-100 was added on top of both the supernatant and the cells (at a 1:1 ratio). Both micro-colonies and substantial cell lysis was observed and thus wells were scraped to incorporate both the supernatant and lysate for escape CFU quantification.

*Finally, the authors may want to comment on why the 'escape' numbers are so high between the THP1 vs the primary cells.*

 There are multiple potential reasons why there was variation between the recovered escape CFU from primary and the THP1 macrophages. Importantly, considerable differences exist between the immortalised THP1 macrophage cell-line and the primary macrophages. For example, a broader repertoire of chemokine and cytokine responses in primary macrophages  could contribute to more efficient killing by the primary cells and thus lower viable recovered CFU.

# Peer review: Reviewer response for version 1

**DOI:** 10.21956/wellcomeopenres.17782.r42336

**Published:** 2021-02-08

**Authors:** Julie A. Morrissey

**Affiliations:** 1 Department of Genetics and Genome Biology, University of Leicester, Leicester, UK

This is a very clearly written interesting and relevant article on the role of two *Staphylococcus aureus* lipases that are secreted into the extracellular milieu. Previous studies have suggested that these lipases may interfere with the function of innate immune cells but no previous articles have investigated the direct role of the lipases. This article describes the phenotypic analysis of a *S. aureus *double lipase mutant and recombinant lipase proteins. There were no differences in the survival between the wild type and mutant strains with human whole blood or neutrophils and no effect on phagocytosis, intracellular survival, or escape from both human primary and immortalised cell line macrophages. Therefore, it was concluded that *S. aureus* lipases do not inhibit bacterial killing mechanisms of human macrophages, neutrophils, or whole blood. The article gives a very clear background of the literature of *S. aureus* lipases in innate immunity. The study design is logical and uses a series of well-established phenotypic assays that are well described and use appropriate controls and are shown to be statistically reproducible. There are no major issues to address.

Is the work clearly and accurately presented and does it cite the current literature?

Yes

If applicable, is the statistical analysis and its interpretation appropriate?

Yes

Are all the source data underlying the results available to ensure full reproducibility?

Yes

Is the study design appropriate and is the work technically sound?

Yes

Are the conclusions drawn adequately supported by the results?

Yes

Are sufficient details of methods and analysis provided to allow replication by others?

Yes

Reviewer Expertise:

Staphylococcus aureus, pathogenesis, host-microbe interactions

I confirm that I have read this submission and believe that I have an appropriate level of expertise to confirm that it is of an acceptable scientific standard.

# Peer review: Reviewer response for version 1

**DOI:** 10.21956/wellcomeopenres.17782.r41781

**Published:** 2020-12-23

**Authors:** Francis Alonzo III

**Affiliations:** 1 Department of Microbiology and Immunology, Stritch School of Medicine, Loyola University Chicago, Maywood, IL, USA

In this Research Article, Sargison *et al.* test if the *S. aureus *secreted lipases, Lip1 and Lip2, interfere with the phagocytic killing mechanisms of innate immune cells. Prior work had suggested there might be potential effects on immune cell phagocytosis/killing on account of morphological changes that occurred after the treatment of granulocytes with lipases. Additional work has also demonstrated a role for lipases in interference with immune signaling on account of lipolysis of the lipopeptide ligands of TLR2. No studies to date have directly tested the role of lipases in phagocytic killing or escape from innate immune cells. The authors use a series of well-executed in vitro survival assays with whole blood, primary human neutrophils, monocyte-derived macrophages, and THP-1 differentiated macrophages to monitor the survival of WT *S. aureus *and an isogenic *lip1/lip2 *deletion mutant +/- recombinant Lip1/Lip2 over time. They find that lipases do not have a discernable effect on phagocytic killing or bacterial escape in the assays used. The methods contain sufficient detail, and the results are clear and unambiguous. Incidentally, the work also closely resembles unpublished observations made in our lab with murine neutrophils and macrophages, thus the data are further bolstered by similar outcomes among multiple groups. Overall, this work provides important information related to the roles of lipases in host-microbe interactions. I have no substantive criticism of this solid study.

Is the work clearly and accurately presented and does it cite the current literature?

Yes

If applicable, is the statistical analysis and its interpretation appropriate?

Yes

Are all the source data underlying the results available to ensure full reproducibility?

No source data required

Is the study design appropriate and is the work technically sound?

Yes

Are the conclusions drawn adequately supported by the results?

Yes

Are sufficient details of methods and analysis provided to allow replication by others?

Yes

Reviewer Expertise:

S. aureus pathogensis, host-microbe interactions, immune response to infection.

I confirm that I have read this submission and believe that I have an appropriate level of expertise to confirm that it is of an acceptable scientific standard.
